# Adverse childhood experiences and health among indigenous persons experiencing homelessness

**DOI:** 10.1186/s12889-020-10091-y

**Published:** 2021-01-07

**Authors:** Eric Smith, Katrina Milaney, Rita I. Henderson, Lyndon Crowshoe

**Affiliations:** 1 University of Calgary, 3280 Hospital Dr NW, Calgary, AB T2N 4Z6 Canada; 2 University of Calgary, 3330 Hospital Dr NW, Calgary, AB T2N 4N1 Canada

**Keywords:** Indigenous, ACEs, Health, Health care, Mitigation, Measurement

## Abstract

**Background:**

Current literature has established that adverse childhood experiences (ACEs) are associated with the onset of a variety of physical, mental, and behavioural illnesses. However, there are few studies that have thoroughly examined this association in low-income or marginalized groups.

**Methods:**

To address this knowledge gap, this study used self-reported data on childhood experiences and adult health outcomes in a sample of 91 Indigenous persons experiencing homelessness. While the primary focus of the study was to assess the relationship between ACEs and health status, we also assessed reports on use and perceptions of health care services to test for potential illness-mitigating factors.

**Results:**

Results indicated that reported number of ACEs was significantly associated with reported levels of mental illness (*p <* .001, *d =* 1.12). Significant associations were not observed for physical illness or patterns of substance use. We also found that the number of reported ACEs was significantly correlated with the number of formal health care services that an individual used (*r =* 0.32).

**Conclusions:**

Our results reveal that the relationship between ACEs and adult illness is not as deterministic as the current literature suggests. Access to formal health care services may allow individuals to mitigate their adverse health, thereby eliminating some of the effects of ACEs. Conversely, current tools used to measure ACEs may not translate to an Indigenous population, which speaks to a need to revise ACE related surveys to include additional adversity categories.

**Supplementary Information:**

The online version contains supplementary material available at 10.1186/s12889-020-10091-y.

## Background

### Childhood adversity and adult health

A thorough understanding of an individual’s childhood adversity is crucial to understanding their health status in adulthood [1] The more adversity a child experiences, the more likely they are to develop acute or chronic illness [2–4]. These illnesses can manifest themselves in an individual’s physical health, mental health, or propensity for substance abuse [5–8]. As such, having a comprehensive understanding of childhood adversity helps health care providers accurately predict an individual’s overall well being.

Childhood adversity is defined as having experienced abuse, neglect, or household dysfunction before turning 18 years old [3, 9]. This adversity is unique in that it is especially consistent. Since most children do not have the economic or intellectual resources to live independently from their caregivers, they are subject to the environments their caregivers provide for them. If a caregiver is creating adverse experiences for a child, it is likely that this child will continue to experience this same adversity in the future [10, 11]. Childhood adversity is also unique because it occurs during periods of high neural plasticity. The more adversity an individual experiences during these critical periods, the less likely they are to adequately develop physiologically or psychologically [12, 13].

### The ACE score

The Adverse Childhood Experience (ACE) questionnaire is a tool that was designed to systematically typify and quantify childhood adversity. An individual’s ACE score is determined by the summation of affirmative responses given for ten questions, each of which asks the respondent about a particular type of adversity experienced before their 18th birthday (i.e., abuse, neglect, and household dysfunction). ACE scores close to ten indicate a high level of childhood adversity, while scores close to zero indicate a low level of childhood adversity.

The efficacy of the ACE score was first demonstrated in the seminal ACE Study held at the Kaiser Permanente clinic in San Diego, California [3]. This study administered the ACE questionnaire to 17,500 participants, most of who identified as being non-marginalized and middle-class individuals. The authors concluded that an individual’s ACE score was positively associated with a variety of physical/mental illnesses and addictions in adulthood. These conclusions have been corroborated by more in-depth analyses of the ACE Study dataset [2, 14] and have been replicated by several authors that used samples with different demographic characteristics [15–17]. This reliability has led the ACE score to become the most frequently used measurement for childhood adversity.

### Four or more

Although high ACE scores are associated with an increased risk of illness, there is a general consensus that this risk eventually plateaus [8, 18]. Specifically, it is suggested that individuals with ACE scores of “four or more” are all similarly high-risk for developing adverse adult health. While it is true that most studies have found negligible differences in health risks for individuals with high ACE scores, the vast majority of current research consists of secondary analysis of the ACE Study or uses samples that are largely made up of non-marginalized, middle-class individuals [18]. This inordinately homogenous sampling is problematic because non-marginalized, middle-class individuals experience less childhood adversity than almost any other group [19, 20]. Therefore, the four or more conception may be attributable to sampling that favours those with lower ACE scores, rather than a reflection of the actual nature of the relationship between ACEs and health.

The few ACE studies that have been conducted on marginalized and low-income groups tend to conclude that these populations have higher ACE scores and higher rates of illness than the general population [21–24]. While this information is useful from a demographic standpoint, it does little to remediate the adversity or illnesses themselves. Garnering information in regards to how these high ACE scores and illnesses interact and the implications thereof is crucial for proper preventative health interventions in the future.

#### ACE and indigenous peoples

Several Canadian researchers have examined the relationship between childhood trauma and health and social issues for Indigenous peoples. Muir [25], found that Indigenous youth in the justice system had higher ACE scores than non-Indigenous youth. Hamdullahpur, Kaha, Jacobs and Gill [26] found that Indigenous women with childhood trauma had high rates of suicide attempts and Patterson, Moniruzzaman and Somers [27] found that high ACE scores led to high rates of physical and mental health issues and substance use in Indigenous adults. At the time of our study we could find no Canadian studies that focussed solely on the relationship between high ACE scores and homelessness for Indigenous adults, nor could we find a study that examined Indigenous peoples’ experiences trying to access services for their health needs.

## Objectives

Our study assesses the factors that facilitate the associations between childhood adversity and adult illness. We used a sample of Indigenous persons experiencing homelessness. This sample was chosen because its members represent a marginalized, low-income population that tends to display high rates of adversity and illness. To address individual factors, we sought to determine the nature of the relationship between ACEs and adult health in our sample. To address systemic factors, examined if ACEs are associated with particular uses and perceptions of health care services. This study builds on the ACE literature to date that shows a relationship with childhood trauma and health and social issues in adulthood but with a particular focus on Indigenous adults experiencing homelessness.

## Methods

### Participants

The sample was drawn from the 2016 Calgary Recovery Services Task Force (CRSTF) study on homelessness and substance use. Our sample is comprised of 91 of the total 300 participants in the CRSTF study. The 91 participants were chosen as theyself-identified as Indigenous and lived in Calgary, Alberta.

Data were collected for the CRSTF study by convenience sampling at homeless emergency shelters (Calgary Drop-in Centre, Calgary Alpha House, and Inn from the Cold) and informal communal living areas (i.e., camps). Eligible individuals had to be at least 16 years old, currently experiencing an episode of homelessness lasting more than six consecutive months or reported having experienced at least four episodes of homelessness in the last 2 years. All participants were asked for consent before completion of the survey and given $25 for their participation.

### Materials

The CRSTF study was conducted using an 88 question multiple-choice survey filled out by hand. The wording and layout of the questions were based on a similar survey used in Edmonton in 2015 that assessed homelessness and health needs related to substance use [28]. Ten questions were used to determine the ACE score, each of which asked participants, “Prior to your 18^th^ birthday, did you experience …” (see Additional file 1 for full ACE questionnaire) and participants were asked to respond either “yes” or “no”. The remaining questions were organized into four domains including socio-demographics, drug use and experiences of harm including physical or mental violence because of substance use, health status, and experiences accessing services like treatment program, housing and health care. Questions contained between 4 and 24 response options. Questions with few options tended to be Likert scales, and questions with many options tended to be for enumerating types of illness or substance use.

ACE scores were determined by allotting one point for each “yes” response to a listed adverse childhood event in the ACE questionnaire, for a sum maximum score of 10. After all of the responses were recorded, cleaned, and matched with their respective participants, the dataset was transferred to the SPSS 24 software for analysis. All analyses were conducted using an alpha of 0.05 corrected to 0.0083 with Bonferroni’s correction.

### Procedure

Analysis was divided into two main sections, each of which had three subsections. The first section compared ACE scores to health status, which had the subsections of: physical health, mental health, and patterns of substance use. All of these analyses were conducted using independent samples t-tests. To create the independent health status samples for each subsection, the dataset was divided into relative “high” and “low” groups, which used diagnosed health status as well as reported behaviours as qualifiers (see Table 1). For physical health, participants in the “high negative physical health” reported having three or more diagnosed physical illnesses or disabilities and reported their stress levels as “high” or “very high”. Conversely, individuals in the “low negative physical health” group reported having two or fewer diagnosed physical illnesses or disabilities with reported stress levels of “none”, “slight”, or “average”. In the mental health subsection, participants were placed into the high group if they reported having considered or attempting suicide in their lifetime and having one or more diagnosed mental illness or disability. Therefore, participants were placed into the “low negative mental health” group if they reported never having considered or attempting suicide and having no diagnosed mental illness or disability. Lastly, in the patterns of substance use subsection, participants in the “high substance use” group reported consuming two or more illicit drugs in the last 6 months and consuming more than six alcoholic beverages on one occasion every 7 days or less. Participants in the corresponding low group reported consuming less than two illicit drugs in the last 6 months and consuming more than six alcoholic beverages on one occasion no more frequently than monthly.

**Table 1:** Health Status Categories

Health/Illness	*n*	Description
Physical health		
High	30	≥ 3 diagnosed physical illnesses/disabilities stress = “high” or “very high”
Low	61	< 3 diagnosed physical illnesses/disabilities stress = “none”, “slight”, or “average”
Mental health		
High	39	≥ 1 diagnosed mental illness/disability considered or attempted suicide in lifetime
Low	52	no diagnosed mental illnesses/disabilities never considered or attempted suicide in lifetime
Substance use		
High	36	used ≥ 2 illicit drugs in last 6 months consumes ≥ 6 alcoholic beverages at one time weekly
Low	55	used < 2 illicit drugs in last 6 months consumes ≥ 6 alcoholic beverages at one time no more frequently than monthly

The second section of the analysis compared ACE scores to health care use, which contained the subsections: formal health care use, informal health care use, and perceptions of health care. Formal health care use was analyzed using a linear regression in which ACE scores were compared against the number of different formal health care services an individual accessed within the last year. Informal health care use and perceptions of health care were analyzed using independent samples t-tests. For these tests, grouping participants by their reports of having or not having access to informal health care or adequate health care, respectively, created the independent samples.

## Results

### Demographics

The average age of our sample was 42.35 years, with a mean time spent in homelessness of 7.75 years (this mean is highly conservative; value was calculated by equating “11+ years experiencing homelessness” to 12 years). The mean ACE score was 6.06, and 18% of participants fell below the “four or more” demarcation. With regards to the health status and health care use questions, at least one third of the participants were in the “high” category for each illness measure (Table 1). Additionally, approximately one quarter of the sample reported having no access to informal health care services (i.e., social supports) and 55% of the sample reported having not received enough care from at least one formal health care service. Additional demographics can be found in Table 2.

**Table 2:** Demographics *N* = 91

Characteristic	*n*	%
Gender		
Men	56	62
Women	34	37
Other	1	1
Heritage		
First Nations/Aboriginal	67	74
Metis	22	24
Inuit	1	1
Other	1	1
Number of Times Experiencing Homelessness		
Once	14	15
Twice	14	15
Three Times	6	7
Four Times	12	13
Five Times	10	11
Greater than Five Times	35	39
Length of Time Experiencing Homelessness (Cumulative)		
6–11 Months	2	2
1 Year	1	1
2–3 Years	18	20
4–5 Years	13	14
6–7 Years	9	10
8–9 Years	6	7
10–11 Years	10	11
> 11 Years	32	35
Residential School Exposure		
Family Member Attended	57	63
Participant Attended	18	20
Participant was in Foster Care as a Child		
Yes	67	74
No	24	26
Marital Status		
Married	2	2
Divorced	10	11
Widowed	1	1
Single	57	63
Common Law	16	18
Other	5	5

### ACEs and health status

Independent samples t-tests on ACE scores and health status revealed no significant difference in mean ACE score for participant’s physical health or patterns of substance use. Participants that reported low levels of physical illness (*M =* 5.98, *SD =* 2.70) had ACE scores that were not significantly different from participants that reported high levels of physical illness (*M =* 6.23, *SD =* 2.33), *t*(89) = 0.43, *p* = .666. Moreover, mean ACE scores between participants that reported low levels of substance use (*M =* 5.87, *SD =* 2.74) and those who reported high levels of substance use (*M =* 6.36, *SD =* 2.31) were not significantly different, *t*(89) = 0.88, *p* = .379. The assumption of equal variances was met for both physical health, *F =* 1.19, *p =* .278, and substance use, *F =* 2.42, *p =* .123 according to Levene’s Test.

**Fig. 1:**
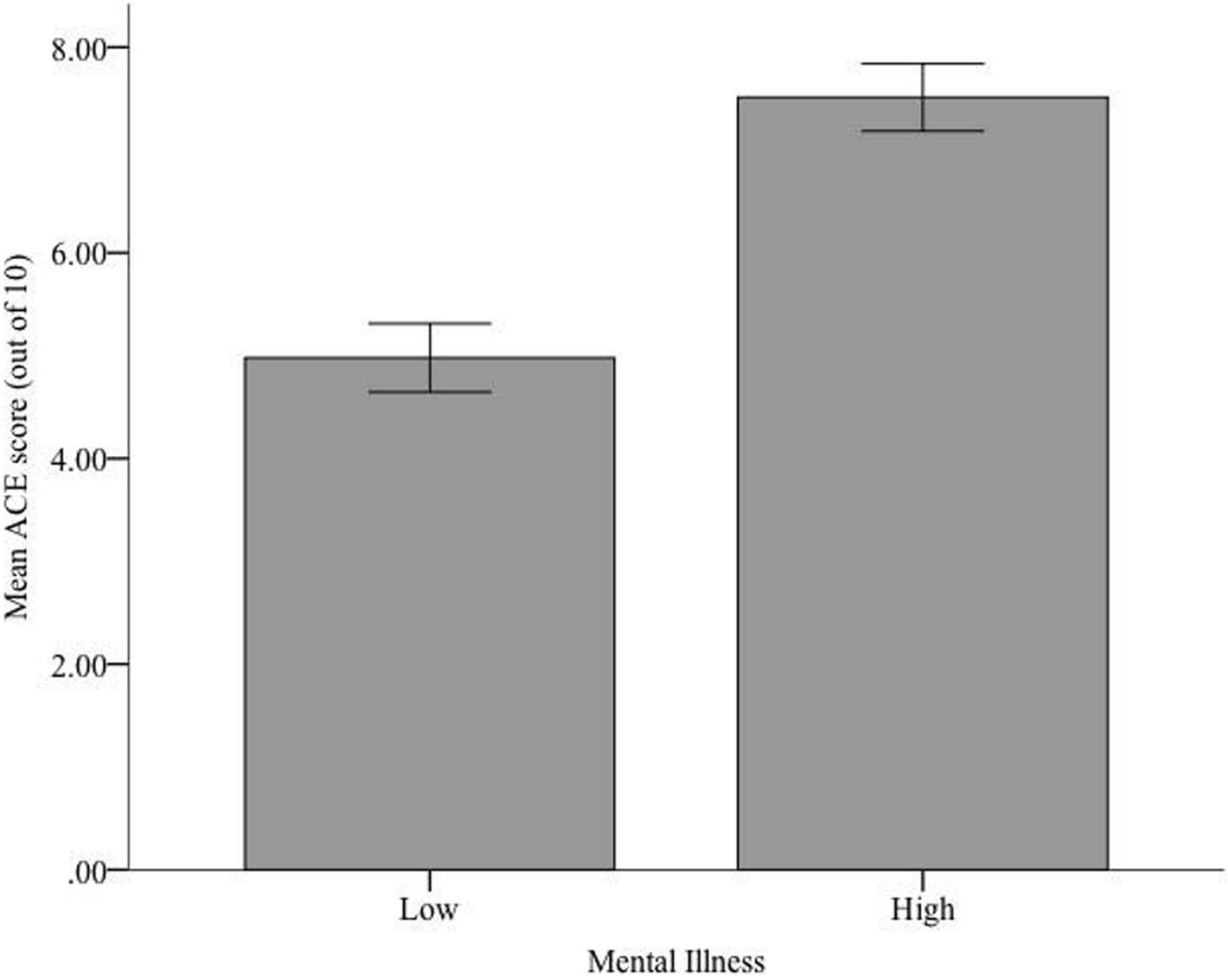
Mean ACE score of each level of reported mental illness. Error bars represent standard error for one standard deviation

These same tests demonstrated that there was a significant difference in mean ACE score for participant’s reported mental health, *t*(89) = 5.30, *p* < .001, *d* = 1.12. More specifically, as Fig. 1 illustrates, participants that reported low levels of mental illness (*M =* 4.98, *SD =* 2.40) had ACE scores that were significantly lower than those of participants that reported high levels of mental illness (*M =* 7.51, *SD =* 2.04). Using Levene’s Test, the equal variances assumption was met, *F =* 1.75, *p =* .190.

### ACEs and health care use

**Fig. 2:**
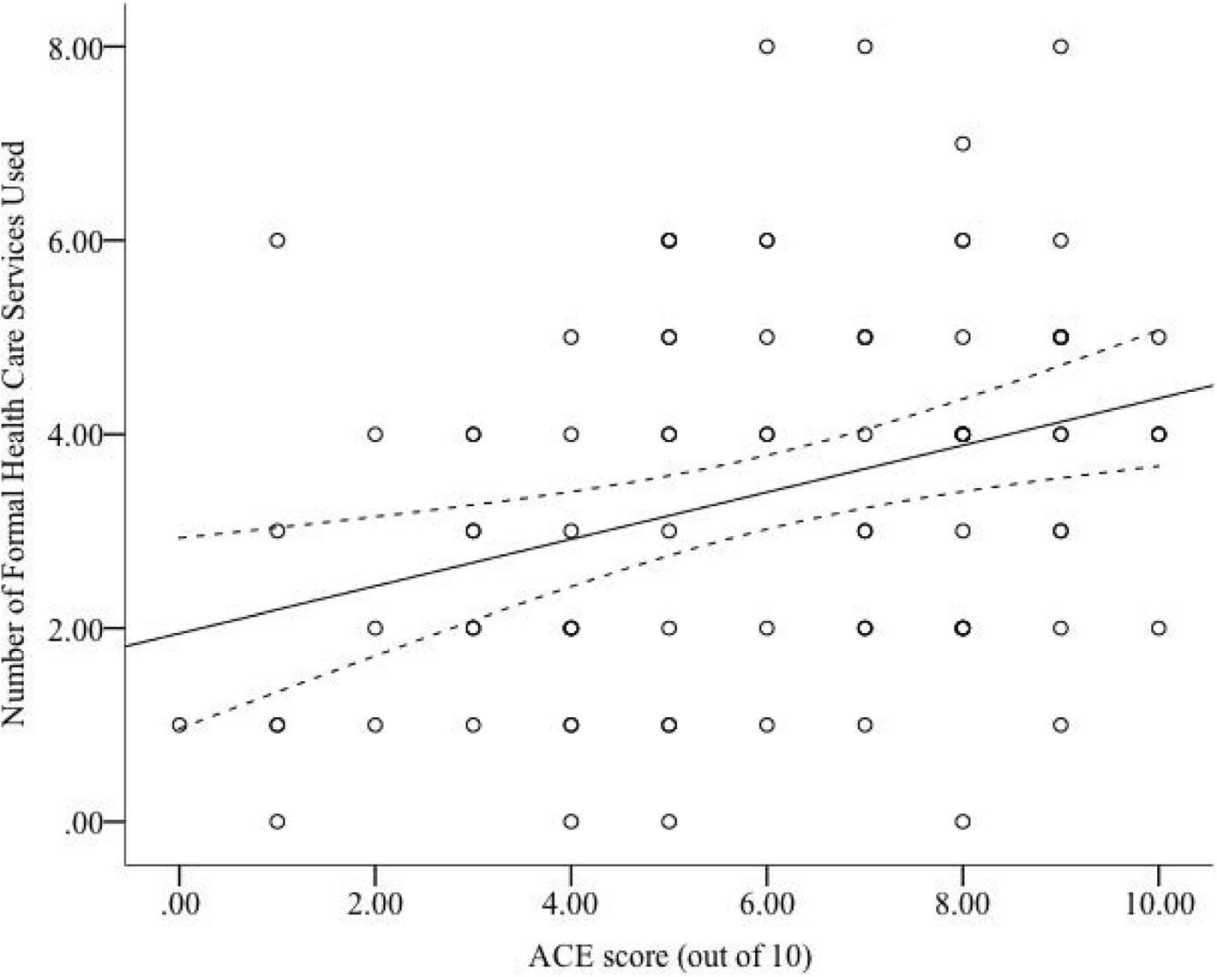
The effect of reported ACE scores on the number of formal health care services used. Dashed lines represent the 95% confidence interval

As can be seen in Fig. 2, the linear regression analysis determined that there was a significant positive association (*r =* 0.32, *p =* .002) between ACE score and participant’s reported use of formal health care services. In other words, participants with high ACE scores tended to report using a greater variety of formal health care services than participants with low ACE scores. ACE score accounted for 10.5% of the variance in participant’s use of health care services. This same effect was not observed for the use of informal health care (i.e., social supports). Using an independent samples t-test, it was determined that the mean ACE scores for individuals that reported having access to health-promoting social supports (*M =* 5.79, *SD =* 2.53) were not significantly different from the ACE scores of individuals that did not have these social supports (*M =* 6.89, *SD =* 2.58), *t*(89) = − 1.75, *p* = .083. Equal variances were found using Levene’s Test, *F =* 0.17, *p =* .678.

### ACEs and health care perceptions

An independent samples t-test on mean ACE score and perceptions of health care revealed no significant effect, *t*(89) = 2.24, *p* = .032 (Levene’s test revealed unequal variances (*F =* 5.26, *p =* .024), so a more conservative *p*-value was used). In other words, mean ACE scores for participants that reported not receiving enough health care from at least one formal service (*M =* 6.60, *SD =* 2.21) were not significantly different from the ACE scores of participants that reported having received enough health care from the formal services they accessed (*M =* 5.42, *SD =* 2.85).

## Discussion

### Implications

#### Normality of adversity and illness

Our study demonstrates that different populations may define “normal” levels of adversity and illness differently. In the current ACEs literature, having ACE scores of “four or more” is the exception rather than the rule. This observation is largely due to the fact that current literature tends to use samples of individuals who experience little adversity. For example, studies with a predominately white, middle class sample, report as few as 6% of participants who experienced four or more ACEs [3]. Comparatively, only 18% of the individuals in our sample had ACE scores below four. Supposing our results reflect patterns in the actual population, it appears to be the case that most homeless Indigenous individuals tend to have experienced high levels of childhood adversity.

As a further point of comparison, the mean ACE score of the 209 non-indigenous participants in the original CRSTF sample was 3.70 [29]. As such, individuals belonging to an Indigenous group tended to experience at least two more types of abuse, neglect, or household dysfunction than their non-indigenous counterparts. Therefore, “normal” levels of adversity are defined differently not only for populations of different income echelons as shown in the Felitti study [3], but also for homeless populations of different cultural backgrounds. .

It should also be noted that the qualifications for “high” and “low” illness (Table 1) were more severe than the metrics used in most other ACE studies. Following the same methods as most other ACEs literature (i.e., defining “low” levels of illness as the absence of most if not all illness) would have made it impossible to conduct statistical comparisons because there were almost no participants that reported having no illnesses. This illustrates that our sample not only has a higher rate of childhood adversity than other populations, but also experiences a higher rate of illness (potentially attributable to the higher rate of childhood adversity).

#### Formal health care’s mediating role

Supposing that formal health care services are, in fact, effective at ameliorating illness, our results could indicate that individuals are able to reduce the severity of their physical illnesses and substance use issues associated with their ACEs if they had access to formal health care. Since most other ACEs literature suggests that ACEs increase an individual’s likelihood of developing physical illness or substance abuse habits, we assume that this explanation holds true for our sample. Given that individuals in our sample with high ACE scores had a tendency to use a large number of formal health care services, these services may have actually facilitated the association between ACEs and health.

Although our participant’s ACE scores did not significantly differ between those who were and were not satisfied with formal health care services, it is worth noting that more than half of our sample reported not having received enough formal care. Even if ACE score is not associated with these perceptions, the potential for more than one half of a group of individuals to leave health care without being properly cared for is disconcerting.

#### ACE score reliability

It has already been suggested that the current ACE questionnaire is limited in that it fails to account for common childhood adversities such as family financial problems, loss of a family member, food insecurity, and cultural norms or social stigmatization [20, 30]. These exclusions are especially important to consider in Indigenous samples. Although many Indigenous people are subjected to severe adversities such as residential schools or inter-generational trauma, these factors are not considered to be ACEs. This may be one possible explanation for why ACE scores were not predictive of physical health and substance use in our study.

### Limitations

One of the main limiting aspects of the study was the qualification criteria used to determine a participant’s illness. Specifically, the decision to only use diagnosed illnesses as a measure of health status may have resulted in a spurious association between ACEs, health status, and health care use. The more health care services that an individual uses, the more likely they are to be diagnosed with an illness. As such, it may have been the case that individuals with high ACE scores were reporting high rates of illness because these individuals were seeing a large number of different health care practitioners, who could potentially diagnose the individual. Furthermore, there is a possibility that many of the illnesses reported by respondents were actually the result of compounding health issues rather than ACEs. In other words, individuals may have displayed physical illnesses due to the severity of their mental illness or substance use.

Another limitation of our study is the ACE score itself. While the previous section already demonstrates that the ACE score has limitations in terms of the types of adversity it assesses, it should also be noted that the administration of the ACE questionnaire has several flaws as well. The most prominent of these flaws is the fact that the ACE questionnaire is administered to adults. As time between an experience and recalling said experience increases, accuracy of recall decreases [31]. Given that the mean age of our sample was 42.35 years old and participants were being asked to recall experiences before the age of 18, it is reasonable to assume that reports on ACEs were not entirely accurate.

Our study may also have had high rates of underreported illness due to an acclimatization effect. Since most of our participants were chronically homeless, it may be the case that these individuals have been experiencing their respective illnesses for many years. As such, when the participants were asked about their current health status, they may have considered their illness to be trivial and chose not to disclose it. These individuals may have also decided to underreport their illnesses due to stigma surrounding particular health issues or a learned pattern of behaviour to not report adverse health because it leads to unwanted attention or care (i.e., social workers/government intervention).

Finally, in our sample, 62% were men; literature has suggested that women are more likely to utilize healthcare services than men [32]; however, sex was not accounted for in our analysis. This would be an important consideration in future analyses.

### Future directions

Despite the fact that current literature suggests that ACEs are a significant factor in explaining an individual’s propensity for illness in adulthood, there is still little being done in health care to account for or monitor ACEs [9, 13, 32, 33]. Our study corroborates this knowledge not only by suggesting that ACEs are associated with adult illness, but also by suggesting that health care services can mitigate the severity of this illness. To further improve upon the effects that formal health care services currently have on illnesses, we suggest two alterations. First, we suggest introducing formal ACE training and ACE screening into health care. While we acknowledge that most health care practitioners are required to undergo brief online modules on complex patients, this is far from rigorous training on trauma-informed care. A more in-depth trauma-informed care training requirement will not only increase health care providers’ awareness of ACEs and the discrepancies in “normal” levels of adversity between populations, but will also aid in the creation of a more holistic approach to health care that understands the impacts of inter-generational and individual-level trauma. Second, our findings suggest that formal health care services need to direct more resources towards mental health care. Supposing that our observed association between ACEs and mental illness is the result of a lack of formal mental health care, introducing more comprehensive mental health care services may result in effective mitigation of even more ACE-related illnesses in the future. Moreover, a more comprehensive mental health care system would reduce the likelihood of co-morbidities and/or physical illness resulting from severe mental illness.

We also suggest that the ACE score needs to be expanded to reflect more types of adversity. This expansion would involve including factors such as socioeconomic status, social stigmatization, and systemic oppression such as residential schools. There are already several studies that have identified some of these important adversity factors that are currently missing from the ACE score, and we believe that our study illustrates this gap. The completion of a more comprehensive and/or group-specific ACE questionnaire should be accompanied by rigororous reliability testing that assesses the associations between ACEs and health status in several different permutations of socioeconomic status and social strata. Finally, the Benevolent Childhood Experiences Scale (BCE) “assesses the presence of 10 favorable childhood experiences reflecting love, predictability, and support, and yields a sum total score out of 10, similar to the ACEs scale”. In a study by Merrick, Narayan, DePasquale and Masten [34] use of the BCE in a sample of homeless parents showed that assessing positive early life experiences predicted lower odds of psychological distress. Use of the BCE scale could provide a related, yet different assessment that is focussed on strenghts that could be helpful in better understanding and responding to the health needs of Indigneous adults experiencing homelessness. The effects of such improved measurement systems could be further strengthened by mandating that ACEs and/or BCE’s are added to all electronic health records, and taken into account when caring for any and all patients.

## Supplementary Information

**Additional file 1.**

## Data Availability

The datasets used and/or analysed during the current study is available from the corresponding author on reasonable request.

## Abbreviations

ACE: Adverse Childhood Expereinces
CRSTF: Calgary Recovery Services Task Force
