# Supplementary material for: Adverse childhood experiences and health among indigenous persons experiencing homelessness
Source: BMC Public Health. 2021 Jan 7;21:85. doi: 10.1186/s12889-020-10091-y (PMC7791826; doi:10.1186/s12889-020-10091-y)
Supplement: Supplementary file 1 — Additional file 1. [file 12889_2020_10091_MOESM1_ESM.docx]

Appendix A

The ACE Questionnaire

***Prior to your 18^th^ birthday:***

1. Did a parent or other adult in the household **often or very often**…

Swear at you, insult you, put you down, or humiliate you?

**or**

Act in a way that made you afraid that you might be physically hurt?

Yes No If yes enter 1 ________

2. Did a parent or other adult in the household **often or very often**…

Push, grab, slap, or throw something at you?

**or**

**Ever** hit you so hard that you had marks or were injured?

Yes No If yes enter 1 ________

3. Did an adult or person at least 5 years older than you **ever**…

Touch or fondle you or have you touch their body in a sexual way?

**or**

Attempt or actually have oral, anal, or vaginal intercourse with you?

Yes No If yes enter 1 ________

4. Did you **often or very often** feel that …

No one in your family loved you or thought you were important or special?

**or**

Your family didn’t look out for each other, feel close to each other, or support each other?

Yes No If yes enter 1 ________

5. Did you **often or very often** feel that …

You didn’t have enough to eat, had to wear dirty clothes, and had no one to protect you?

**or**

Your parents were too drunk or high to take care of you or take you to the doctor if you needed it?

Yes No If yes enter 1 ________

6. Was a biological parent **ever** lost to you through divorced, abandonment, or other reason?

Yes No If yes enter 1 ________

7. Was your mother or stepmother:

**Often or very often** pushed, grabbed, slapped, or had something thrown at her?

**or**

**Sometimes, often, or very often** kicked, bitten, hit with a fist, or hit with something hard?

**or**

**Ever** repeatedly hit over at least a few minutes or threatened with a gun or knife?

Yes No If yes enter 1 ________

8. Did you live with anyone who was a problem drinker or alcoholic or who used street drugs?

Yes No If yes enter 1 ________

9. Was a household member depressed or mentally ill or did a household member attempt suicide?

Yes No If yes enter 1 ________

10. Did a household member go to prison?

Yes No If yes enter 1 ________
